# Capturing implementation knowledge: applying focused ethnography to study how implementers generate and manage knowledge in the scale-up of obesity prevention programs

**DOI:** 10.1186/s13012-019-0938-7

**Published:** 2019-09-18

**Authors:** Kathleen P. Conte, Abeera Shahid, Sisse Grøn, Victoria Loblay, Amanda Green, Christine Innes-Hughes, Andrew Milat, Lina Persson, Mandy Williams, Sarah Thackway, Jo Mitchell, Penelope Hawe

**Affiliations:** 1 The Australian Prevention Partnership Centre, Ultimo, NSW 2007 Australia; 2 Menzies Centre for Health Policy, School of Public Health, and University Centre for Rural Health, Faculty of Medicine and Health, The University of Sydney, Sydney, New South Wales 2006 Australia; 3 McMaster University, Hamilton, Ontario Canada; 4 The Australian Prevention Partnership Centre, based at the Menzies Centre for Health Policy, School of Public Health, Faculty of Medicine and Health, The University of Sydney, Sydney, New South Wales Australia; 5 New South Wales Office of Preventive Health, New South Wales Ministry of Health, Sydney, Australia; 6 Centre for Epidemiology and Evidence, New South Wales Ministry of Health, Sydney, New South Wales Australia; 7 Health Promotion Service, South Western Sydney Local Health District, Liverpool, New South Wales Australia; 8 Centre for Population Health, New South Wales Ministry of Health, Sydney, New South Wales Australia

## Abstract

**Background:**

Bespoke electronic information management systems are being used for large-scale implementation delivery of population health programs. They record sites reached, coordinate activity, and track target achievement. However, many systems have been abandoned or failed to integrate into practice. We investigated the unusual endurance of an electronic information management system that has supported the successful statewide implementation of two evidence-based childhood obesity prevention programs for over 5 years. Upwards of 80% of implementation targets are being achieved.

**Methods:**

We undertook co-designed partnership research with policymakers, practitioners, and IT designers. Our working hypothesis was that the science of getting evidence-based programs into practice rests on an in-depth understanding of the role programs play in the ongoing system of local relationships and multiple accountabilities. We conducted a 12-month multisite ethnography of 14 implementation teams, including their use of an electronic information management system, the Population Health Information Management System (PHIMS).

**Results:**

All teams used PHIMS, but also drew on additional informal tools and technologies to manage, curate, and store critical information for implementation. We identified six functions these tools performed: (1) relationship management, (2) monitoring progress towards target achievement, (3) guiding and troubleshooting PHIMS use, (4) supporting teamwork, (5) evaluation, and (6) recording extra work at sites not related to program implementation. Informal tools enabled practitioners to create locally derived implementation knowledge and provided a conduit between knowledge generation and entry into PHIMS.

**Conclusions:**

Implementation involves knowing and formalizing what to do, as well as how to do it. Our ethnography revealed the importance of hitherto uncharted knowledge about how practitioners develop implementation knowledge about how to do implementation locally, within the context of scaling up. Harnessing this knowledge for local use required adaptive and flexible systems which were enabled by informal tools and technologies. The use of informal tools also complemented and supported PHIMS use suggesting that both informal and standardized systems are required to support coordinated, large-scale implementation. While the content of the supplementary knowledge required to deliver the program was specific to context, functions like managing relationships with sites and helping others in the team may be applicable elsewhere.

Contributions to the literature

- In clinical medicine, mundane tasks hidden from formal recording processes are thought to underlie the success or failure of efforts to get evidence-based programs into practice.
- In population-level prevention, we show that part of the success of a state-of-the-art electronic information management system accompanying statewide scale-up may be the unreported and extra work undertaken by practitioners and captured in supplementary recording technologies.
- “Informal” recording technologies demonstrate that practitioners generate additional knowledge about implementation in the course of practice as they accommodate local accountabilities alongside “top-down” goals.
- Nimble technologies are needed to respond to emerging knowledge produced by self-organizing systems at scale

## Introduction

The scale-up of effective programs no longer relies simply on passive methods of education or knowledge diffusion. Instead, the focus is on developing purposeful strategies and mechanisms to mobilize the translation and enactment of research knowledge into practice on a large scale. In Australia, a landmark health information technology (IT) system—the Population Health Information Management System (PHIMS)—is currently supporting the largest-ever implementation of evidence-based childhood obesity prevention programs in the country. Funded by the New South Wales (NSW) government, the Healthy Children Initiative (HCI) is a suite of evidence-based policies and programs to address childhood obesity through settings-based approaches [1].

PHIMS supports the large-scale implementation of two of HCI’s flagship programs—Live Life Well @ School and Munch and Move®. Both programs support the implementation of evidence-based practice recommendations from the World Health Organization including that schools and childcare settings create healthy food environments, and that these settings have adequate facilities to support physical activity [2]. Live Life Well @ School targets primary school settings while Munch and Move® targets early childhood services. Both programs are effective in producing settings-based environmental changes that promote healthy eating and physical activity practices [3, 4]. They are achieving high reach at scale with 89% (3348/3766) of childcare services and 81% (2133/2566) of primary schools participating [5]. Ongoing monitoring has shown steady progress in the implementation of practices over time. Over the period of 2012–2015, the proportion of early childhood services serving only water or age-appropriate drinks increased from 33% to 71% [6]. In schools, physical activity at breaks, teacher learning, and development for healthy eating and physical activity and other practices likewise significantly increased [7].

Via a four-way practitioner-policymaker-IT developer-researcher partnership, we had the opportunity to study PHIMS use, and through PHIMS, the implementation of HCI. Because HCI has been delivered and sustained since 2011, we were particularly interested in how information about program implementation has been used and managed for sustained implementation, supported, in part, by the PHIMS system.

There is increasing recognition that understanding how implementers use, adapt, and importantly, create new knowledge in practice is key to understanding why some implementation endeavors are successful while others fail. In clinical settings, previous research has exposed the importance of “hidden work” (i.e., largely unseen work) completed by practitioners that is required to adopt, integrate, and sustain an innovation in practice [8]. The seemingly mundane tasks that often go unnoticed or unrecognized likely underlie the success of implementation endeavors. We set out to understand the extent to which this may also be the case in the delivery of population-level prevention programs. To our understanding, our study is the first of its type. There is little documentation of IT systems in population-health contexts, and what does exist suggests that these systems fail more often than succeed [9]. To our knowledge, PHIMS is rare in that it enjoys sustained use and was recently expanded to include health issues beyond obesity. Studying PHIMS in-depth provides an opportunity to observe the knowledge gathered, used, and generated by practitioners in the day-to-day implementation of a scaled-up program. It is a unique opportunity to explore any unseen work that exists alongside PHIMS use. Insights will be valuable for IT design and uncovering previously undiscerned dynamics of implementation. Specifically, this study aims to examine how practitioners use and create knowledge in the ongoing implementation of obesity prevention programs. Our research objectives were (1) to explore what kinds of information and knowledge are valued by practitioners for implementation and (2) to examine how PHIMS sits alongside other systems used by practitioners to generate and capture such knowledge for implementation.

### Using PHIMS to track program implementation

HCI is delivered by 14 teams of Health Promotion Officers (hereafter referred to as “practitioners”) situated across 15 local health districts (LHDs) funded and supported through the NSW Ministry of Health [5]. Annual health service agreements [10] set out service and performance expectations to ensure the provision of safe, high-quality, patient-centered healthcare services including prevention. Collectively, HCI teams support over 6000 primary schools and early childcare services to achieve a specified number of evidence-based practices as described above and elsewhere [11]. The achievement of these practices constitutes the key performance indicators (KPIs) by which implementation progress and fidelity are monitored and measured. PHIMS was developed to capture this performance data and was designed with a dual purpose: to (1) support the implementation of HCI programs while (2) simultaneously aggregating and reporting data for implementation monitoring and to inform future policy and programmatic decisions.

The HCI performance monitoring approach [11] includes two levels of indicators for assessment: Service Delivery Indicators—i.e., reach, follow-up, and support provided by practitioners—and KPIs—i.e., specified implementation targets for HCI. These two functions are built into the design of PHIMS itself and enable PHIMS to simultaneously serve multiple key user groups:

- Health Promotion Officers: enter data about their day-to-day work and use PHIMS to support the delivery of the programs. They generate the primary data that PHIMS aggregates and reports to other users.
- Managers of HCI teams: monitor achievement of program practices and the activities and impact of their team members.
- The Office of Preventive Health: monitor program implementation, enabling statewide responses to specific targets which need strengthening or quality improvement. These users can access aggregated data for the LHD in real-time. They are unable, however, to access site-level data and notes.
- The Ministry of Health: track the LHD's delivery on KPI targets. Reports generated from PHIMS data are used in performance meetings with LHD Chief Executives.

**Fig. 1:**
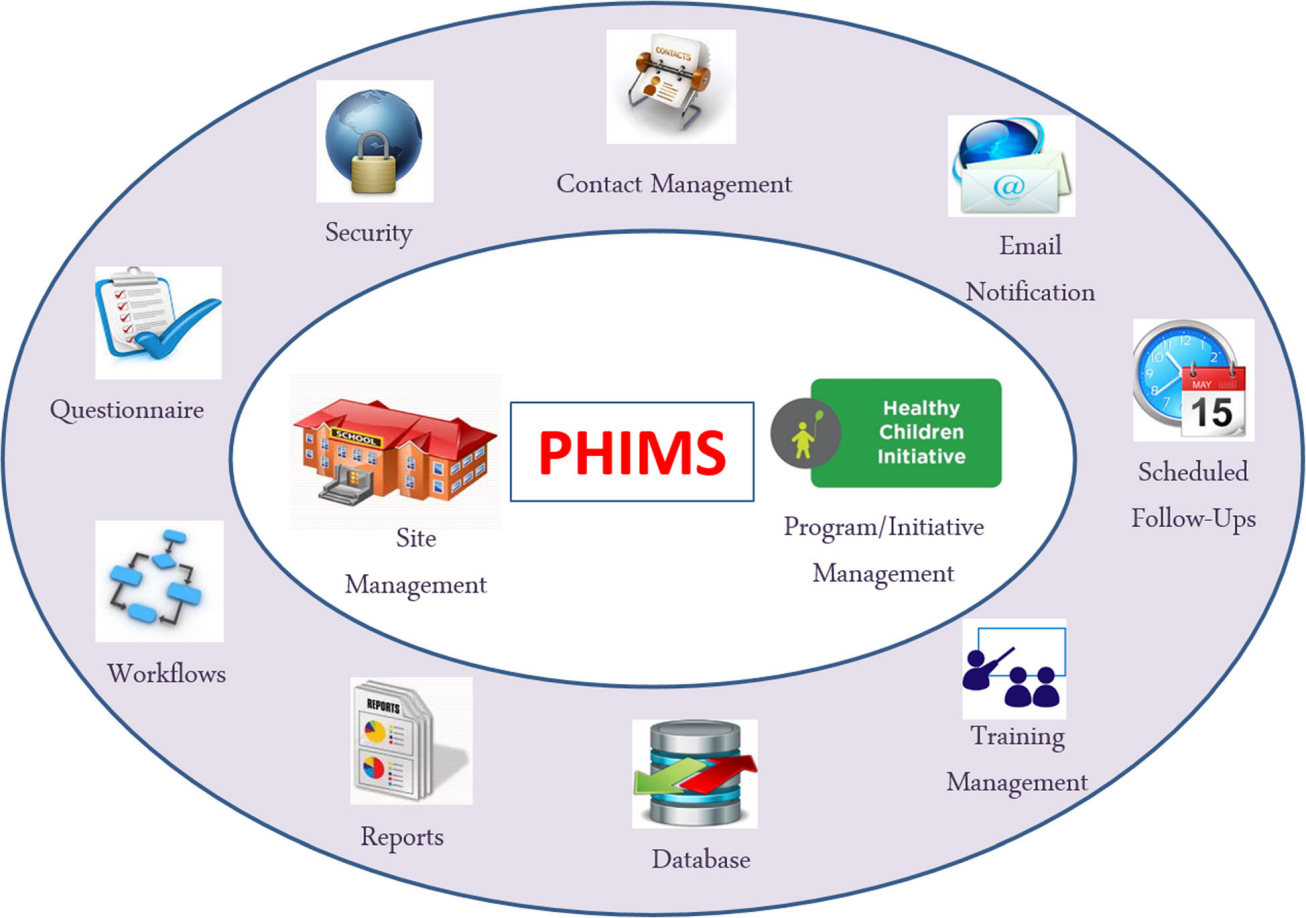
Functions built into the design of the Population Health Information Management System (PHIMS). PHIMS is a dual-purpose system, designed to (1) track and report progress against key performance indicators of the Healthy Children Initiative and (2) provide site management support tools to end-users. For purpose 1, key functions include secure data collection via questionnaires, workflows ensuring timely reporting of data, and the ability to generate reports. For purpose 2, key functions include contact management, email system, scheduling system for tracking follow-ups with sites, training management for site contacts, and a database for filing practice notes

The development of PHIMS and a detailed description was previously reported [12]. Briefly, PHIMS is protected through a series of access control settings that are configured according to the users’ role. Figure 1 provides an overview of PHIMS’ main functions. Users at the practitioner-level are provided an overview of their district’s performance compared against statewide performance. Users access their assigned sites via three main interfaces:

1. General information about each site, e.g., contact persons and training status of staff.
2. Questionnaires about how the sites are performing on implementation targets. Users enter data via a Likert-type survey form. PHIMS aggregates the data and feeds it back via user reports.
3. System-generated reports about training, sites requiring follow-up, practice achievement, or program adoption.

## Methods

This study is part of a larger project, co-produced through a collaboration between researchers, policy-level decision-makers in NSW health, IT designers of PHIMS, and state-level HCI coordinators. The research approach and specific research questions were designed over a period of extensive collaboration between the partners and with the overarching aim of examining how PHIMS intersects with HCI practice [13]. This co-production process continued between the larger research team and partners throughout the currently reported project. The current article uses data from ethnographic field notes collected with all 14 HCI teams in New South Wales.^1^ We used the consolidated criteria for reporting qualitative research (COREQ) guidelines [14] to guide our study and referred to the Standards for Reporting Qualitative Research (SRQR) [15] reporting (for more details, see Additional file 1).

The research was informed by the concept of multi-sited ethnography, in which the “field” is grounded in systems rather than geographic locations [16–18]. In this way, we conceptualized the field as a system of connections between people, places, and objects mediated through and by PHIMS [17]. In each local health district, we adopted an approach consistent with “focused” ethnography [19], characterized by intensive data collection during site visits with practitioners but where contact with sites preceded and extended beyond in-person visits (detailed below). We drew on a range of theories (see [13] for extended list) as sensitizing concepts prior to entering the field to “suggest directions along which to look” [20], and throughout the writing, analysis, and interpretation process. Incorporating theory throughout all aspects of the project reflects an ongoing dialog between theory and practice [21] and enabled us to recognize specific constructs during data collection and initial analysis that related to our guiding research questions while remaining open to emergence.

### Data collection

Three researchers (KC, SG, and VL) undertook ethnographic fieldwork across a period of 12 months (August 2016–2017). One (KC) conducted preparatory work for 1 year prior to entering the field in which she conducted exploratory interviews with project partners, undertook training in PHIMS, and met with multiple sites to design the fieldwork approach with PH. Consistent with a focused ethnographic approach [19], the team of ethnographers immersed themselves in this data, undertook a 3-day intensive collaborative site visit, and engaged in extensive theoretical readings prior to independently entering the field. Each independent field visit was followed by a group debrief and reflexive discussions of the fieldwork experiences.

We shadowed, interviewed, or observed 106 practitioners across all 14 teams. Access to teams and participants varied depending on trust built, the participants’ willingness to host researchers, and interest in the topic. Geography also influenced access in that ethnographers traveling to teams in regional areas spent intensive periods varying from 1 to 5 days. For some of the metro-based sites in closer proximity, researchers were able to selectively schedule visits around activities and meetings over a longer period.

What our focused approach to ethnography lacked in terms of duration in the field it made up for in purposeful selection and engagement with participants and a variety of data sources [21]. The focused nature of visits resulted in intensive engagements where ethnographers worked alongside participants in their offices and accompanied them when they visited schools, workshops, and meetings. Fieldwork activities were documented in extensive field notes providing us with a large quantity of data for the time spent on visits [19]—over 500 pages of typed, single-spaced field notes in the dataset. Other data collected—pre-, during, and post-fieldwork—includes photos (about 65), written program documents and materials (over 100 documents), ad hoc recorded interviews (over 50 h have been transcribed verbatim), and subsequent email discussions with participants (numerous and ongoing). Following site visits, continuous iterations of fieldwork included presenting at statewide health promotion workshops, holding statewide video webinars, returning to LHDs for feedback workshops, and giving seminars to the PHIMS IT design team. As is typical of focused ethnography methods, these engagements enabled researchers to reengage with the ethnographic context long after leaving the field, each time gaining new insights and allowing flexibility to explore emerging issues [21]. This approach differs from other short-duration ethnography types—often called “rapid ethnographies” now widely adopted in computer studies and increasing in healthcare studies [22]—and is typically applied to inform specific practice changes.

The ethnographic research team also consulted and met with the broader co-production partnership team (the authors of this paper) periodically throughout the fieldwork to discuss select, anonymized results from our dataset. In this way, the co-production partnership group acted as “para-ethnographers” whose reflexive awareness of the ethnographic material helped to co-inform our research process [23, 24]. The continuous circulation and reinterpretation of data with research participants and our partners extended the ethnographic “place” beyond the intensive field trips to include processes of data analysis and dissemination [21]. These sharing processes also facilitated reflexive practice among the ethnographers, prompting ongoing consideration of our role, perspectives on the data, and our ability to facilitate change through the research process [25].

### Analysis

The research question guiding this analysis was established with the project partners at the outset of the research project. Other papers in our project (e.g., Loblay V, Conte K, Groen S, Green A, Innes-Hughes C, Milat A, et al: Collaborative friction and knowledge generation: a co-production dialogue within a researcher-policymaker-practitioner partnership examines the value of unreported practice. In preparation) explicitly draw on multiple data sources and adopt a more explicitly reflexive approach to analysis akin to traditional ethnographic approaches. For this analysis, however, we adopted a focused approach to systematically code field note data to answer the guiding research question. Therefore, this analysis is consistent with conventional content analysis [26] from which subsequent codes were developed via a grounded approach. As described fully in our protocol [13], we used NVIVO [27] to develop an overall project codebook to organize and sort data at a high level. Using this codebook, we extracted content from material coded as “tools and methods other than PHIMS that are used to organize, monitor, and structure practice.” This resulted in 142 instances of coded content (ranging from a sentence to multiple paragraphs) from 44 unique field notes. First, we identified the range and type of specific tools or methods used in addition to PHIMS. Next, we identified 24 keywords, mostly names of tools (e.g., excel, diary, reminders) and conducted text searches across the entire dataset to identify additional content previously missed. Two authors (KC and AS) iteratively coded the data to identify what activities the tools are used for in practice. We conceptually organized activities into broader functions of practice and ran cross-coding comparisons to identify the types of tools used for each function. Any discrepancies were resolved through discussion, and difficult or interesting cases were brought to the larger team for discussion and resolution. We presented initial findings to partners and study participants for comment and reflection.

## Results

We observed that PHIMS played a central role in managing information for program implementation. Although the degree to which practitioners and teams used PHIMS varied, all teams used PHIMS to log information about implementation target achievement for sites, to monitor progress towards overarching scale-up implementation targets, and to keep notes about interactions with site contacts. But all teams *also* used informal tools outside of PHIMS to manage program implementation work and to manage new knowledge about implementation. The following excerpt illustrates how PHIMS is used alongside other tools in practice:

The [practitioner] says I have probably seen how [they] all use their own separate Excel spreadsheet to monitor their sites. To her, “that kind of indicates that really that PHIMS doesn’t do everything that we want it to do cause we do keep just a separate [system], I think probably just so it has the most important information that we have so we can just look at it at a glance…without this spreadsheet I would feel a little bit lost with what I’m doing”.*—*Field note from LHD F

### Range of informal tools used in addition to PHIMS

Tools ranged from complex—such as bespoke spreadsheet and database systems designed by the local team—to simple—including standard computer applications and task management tools like email and electronic and physical filing systems. The types of tools observed in practice are described in Table 1. The tools that we observed across most teams were spreadsheets, followed by locally developed templates that practitioners print and take to site visits for notetaking and data collection. Some informal tools existed prior to PHIMS implementation and were adapted over time while others emerged alongside it. The tool used sometimes reflected individual preferences and organization styles and often reflected team approaches to practice. Some teams developed complex spreadsheets or electronically shared files to coordinate work and data entry. Often, multiple tools were used concurrently with PHIMS—a practitioner might have PHIMS opened on one computer screen, email on another, a hardcopy template with notes from a recent site visit, a paper diary, and a spreadsheet tacked to the wall with upcoming deadlines. Informal tools were often used as a complement to PHIMS; for example, spreadsheets were used to manage data downloaded from PHIMS to query specific information. But tools also performed functions that PHIMS was not able to do or could be done more efficiently through another tool. These functions are described in the next section.

**Table 1:** Description of (informal) technologies and tools used alongside a standardized monitoring system

Informal knowledge management tools	Description	No. of LHDs (*n* = 14) in which tool was observed
Backups, hardcopies from PHIMS	Printing forms from PHIMS to use in hardcopy, or to store in another location	7
Bespoke IT system/database	Locally designed IT database	1
Email	Using an email client (e.g., Outlook) externally to PHIMS	8
Filing systems		
Paper filing system	Storing information in hardcopy in a physical location	4
Shared folder or drive	Storing information in digital form on a secured, shared drive	4
Memory	Instances where memory is explicitly described as a way of capturing and managing information	3
Online survey tools	Use of online tools (e.g., survey monkey) to gather, manage, or report information	8
Posted documents	Information that is in hardcopy and posted close-at-hand, at work stations or in offices, for quick access	3
Spreadsheet	Spreadsheets/Excel	12
Task management tools	Systems (e.g., to-do lists, diaries, reminders) to organize and manage tasks	2
Templates	Documents that have been locally developed and are standardized to gather specific types of information	11
Hand written notes and other tools	Not described above; includes handwritten notes	5

Note: *LHD* local health district

### Functions of informal tools

We identified six key functions that informal tools serve in the context of HCI program delivery. These are (1) relationship management, (2) monitoring progress towards target achievement, (3) guiding and troubleshooting PHIMS use, (4) supporting teamwork, (5) conducting an evaluation, and (6) recording work that does not count towards HCI implementation. A single tool was often used for multiple functions and we observed that all the tools identified were used for many inter-connected functions. These are described in Table 2. In Table 3, we provide excerpts of field notes that are exemplars of the functions that informal tools serve; key themes are highlighted in italics below.

**Table 2:** Local health district teams’ use of informal technologies, by type and function

Function	Number of teams using tools, by function	Type of informal tool observed										
		Backups, hardcopy	Bespoke IT system	Email	Filing system: paper	Filing system: electronic	Memory	Online survey tools	Posted documents	Spreadsheet	Task management system	Templates
Relationship management	13	5	1	3	3	4	2	1	2	8	1	10
Monitoring progress towards target achievement	7	5	–	1	1	1	1	4	–	4	–	1
Guiding and troubleshooting PHIMS use	6	3	–	2	–	1	1	–	2	6	2	3
Supporting team work	6	4	–	5	2	3	–	–	–	3	–	1
Conducting evaluation	5	–	–	2	–	–	–	5	–	–	–	–
Recording work that does not count towards HCI implementation	4	–	1	–	–	–	–	1	–	3	–	–

Note: *HCI* Healthy Children Initiative

**Table 3:** Functions that informal technologies perform in practice, themes, and exemplars from ethnographic field notes

Function	Excerpt from ethnographic field notes
Relationship management	
Keeping detailed records of interaction with contacts	[Name] shows me that she keeps a list of all staff and their mail addresses and she will print that out and bring it along, so she can update it if there has been any changes. There is so much turnover in this industry, that she needs to keep track of the changes. – LHD L PHIMS also does not track individuals/teachers as they move around within the LHD – so when they move to a different school or day care setting. This is important to them because these educators have training and relationships with the HP staff, relationships that have taken a long time and work to build. However, PHIMS does not allow you to “move” an educator from site to site within the system. Essentially, you have delete the educator and create a new record for them at a different school… - LHD M
Keeping historical record of relationships with sites and contacts	[Name] also has been responsible for updating their school database. They keep a database separate from PHIMS, with all the data they need for their various projects, it goes back more than 10 years. –LHD D
Capturing subjective impressions and details of relationships with contacts	[Name] says that if she is doing a phone follow-up she will get out the paper file because it is good to know what they are like if she is going to be having a phone conversation. That way she will know if they are “friendly” or whether they are “open” – so if someone is not open to changing the milk KPI, [name] will put that in the paper file and then if she goes to call them. – LHD E Some of the additional information in the spreadsheet is not in PHIMS – for example when recess and lunch is because that is the best time to contact schools, but that is nowhere to record that in PHIMS. – LHD F
Processing experiences with contacts	[Name] says, “you cannot really track things in terms of comments on PHIMS. You can track the numbers” which is why on paper “it’s like a venting thing, I think, writing it on there. That’s where I feel like it’s safe to write if they were rude or something like that. Not that it benefits anything at all… it’s probably the most personal part.” – LHD E
Recording implementation strategies used across multiple sites	PHIMS cannot capture what they do across a whole set of schools because it’s focused on each individual site. – LHD M
Monitoring progress towards target achievement	
Track program adoption and implementation progress in each site	[Name] shows me the Excel spreadsheet that she’s been working on from the PHIMS drill down report she downloaded yesterday. She still has to do a lot of work preparing the Excel spreadsheet to give her the information that she wants. She is trying to find out how the sites are doing on two specific practices so they can figure out who to target in the next round of incentive grants. Last time, they only targeted schools that were not meeting 80% of [implementation targets] – so as to help get the LHD over their [implementation] targets. – LHD I I ask if I can take a photo of her ‘colourful’ excel spreadsheet, which she has filtered. [She] explains her colour-coding system in the excel spreadsheet – where it is easy for her to see which sites are sitting above 80% for ‘program adoption’ so she colour codes them green. Whilst you can see that information in PHIMS, it’s just easier for her to see it this way. She can also see the sites that are under and who “need a bit more support.” – LHD F
Collect contextual information about sites progress towards targets	In [a] spreadsheet, [name] enters some info on the service she just visited, such as “have a great healthy eating program” – this is so she has “a bit more context” when she calls them next time “we would not put this kind of information into PHIMS all the time”. –LHD E
Tracking LHD-level targets in addition to state-level targets	She opens a huge master spreadsheet of all of the schools. Here they record detail including the same scoring that goes into PHIMS. The reason they keep the scores in two places is that PHIMS does not allow them to drill down into the details of the low-performing schools. This LHD has an equity framework that they use to prioritize the sites. -LHD N
Guiding and troubleshooting PHIMS use	
Tips and guides to facilitate accuracy and consistency of data entered into PHIMS	One of the monitors has a small printed list physically attached to the bottom of the screen. The list, she tells me, is a set of bullet points to guide what she should note in PHIMS following a site visit. –LHD F
Planning for deadlines in PHIMS	She keeps track of due dates of her sites in her own excel file because she will start working on contacting them about a month before the scheduled follow-up date [in PHIMS]. – LHD I
Critical information is kept available at a glance or close at hand	He keeps his own colour coding excel spreadsheet open while he calls, it is made so he can see the information he need for all his sites on one page... He also has prints on the wall with an overview of the sites and his contacts. – LHD L She’s got 3 spreadsheets taped to her wall. Each represents a different team members way of organizing the work. They are all different with different columns, colours, and level of detail. In addition, she has folders for each school where she keeps hard copies of correspondence, checklists, etc. - LHD N
Copies of data is kept to safeguard against PHIMS going offline or losing data	“I do not think PHIMS is going to open, which is a bit inconvenient. I can show you the last month’s report but I cannot show you exactly how I extracted it…it’s literally because the limitations of PHIMS mean that we still need to maintain some other information off of PHIMS so I still do not really know the best way of doing it. I think each Health Promotion Officer does it differently.” Informal interview in LHD G
Supporting team work	
Histories of interactions with sites is kept to safeguard against turnover of staff	[Name] says she prefers to use her handwritten notes from today, a spreadsheet of all her sites which she records notes on, and the email as her record. The spreadsheet is saved on a shared drive so this is shared with her colleagues who can access it should she be unavailable or leave for a new position. – LHD H They keep a database separate from PHIMS, with all the data they need for their various projects, it goes back more than 10 years. [Name] will train the officer who will take over from her this afternoon. [Name] is in general informing them about what they need to be aware of while she is away. – LHD D
Team work: strategizing, planning, coordinating activities and providing support	They have their own systems that they keep independent of PHIMs. This includes “a lot of excel spreadsheets.” They save emails exchanged between the HCI team and the schools in a shared folder. If one practitioner is having no progress with a school, they swap schools among the team. They do planning/strategize together as a team. – LHD M The database contains the teachers’ names, when they have attended, and the certificates they have received for attending. It has nothing to do with the HCI program practices, it is just to keep track of the workshop. … The database has got all the information she needs to email them, so she can send invitations and information out directly from here. [Name’s] office staff needs to be able to access this because she asks them to do it for her. – LHD J
Conducting evaluation	
Formative evaluation	[The team hosted] student interns for a few weeks who did a survey monkey with the sites about their general needs and barriers to implementation… As a result of the survey monkey and practice experience, the Health Promotion team identified that a handout/poster with key messages … would be beneficial. – LHD H
Process evaluation	Following the two rounds of workshops, [Name] said they have been able to do “a bit of an evaluation” based on pre and post in terms of the “confidence levels” of cooks and directors. She showed me a print out of the results and said that overall confidence levels have increased. – LHD B She is entering data into survey monkey – these are feedback forms they collected at the end of the workshop…This is a separate system from PHIMS. – LHD M
Recording work that does not count towards HCI implementation	
Documenting interactions with non-program sites or community organizations	As an example of what PHIMS does not do, she tells me that the HP department here is working on some sort of IT system to help track their work with community organisations. It sounds like this would be a database of interactions so that when tobacco goes into a community org, they know if the HCI team has already worked with them, in what capacity, who it was, etc. so they can better coordinate their work. – LHD M The HP officers often see new childcare centres opening in the area. When HP staff notice them, they add them to an “off-record” list at the office, then stop by with [program] information and incentives. However, they do not add the site to the PHIMS system until they agree to come on board. – LHD H

Note: Unless otherwise noted, excerpts are from qualitative field notes, written in the first person by the researchers. Quotation marks denote verbatim quotes from participants

*HP* health promotion, *LHD* local health district, *PHIMS* Population Health Information Management System, *HCI* Healthy Children Initiative

#### Relationship management

The most common function of informal tools (observed across 13 teams) was to manage relationships with key HCI contacts. We observed that all the categories of tools were used to generate and manage knowledge about relationships, including an IT system that had been created by one LHD to manage information about local collaborations. Managing relationships is a key part of implementing HCI programs. PHIMS has inbuilt contact management features for this purpose, but informal tools enabled users to capture more bespoke information. Practitioners used tools outside of PHIMS to *keep detailed records of interactions with contacts* at sites, including a range of diverse stakeholders. Almost all teams used spreadsheets to track details of sites and stakeholders, record the number of contact attempts they make with a site, and/or to list materials given to contacts. Sometimes, relationships between practitioners, sites, and contacts extended back before the HCI program and PHIMS existed, and informal tools were needed to keep a *historical record of relationships with sites and contacts*.

Informal tools captured *subjective impressions and details of the relationship with contacts*. Examples include notes about a stakeholder who is resistant or not ready to make changes to policy and practice, capturing the pet projects of a principal or teacher that could be used to tailor messaging and strategies, or recording the best time of the day to contact a site. Practitioners captured this information via handwritten notes or using templates they developed specifically for this purpose. Informal tools allowed practitioners to write freely, without concern for how their initial impressions might be judged by another in a formal, standardized system like PHIMS. One practitioner explained how writing her initial notes on paper allowed her to *process her experience with the contact* and “vent” about frustrations experienced before entering a formal note in PHIMS.

Practitioners used informal tools to capture more details about implementation than PHIMS can collect, such as specific information about local contexts. Creating good relationships with site contacts was important to practitioners but can take a long time. Some practitioners wanted to track this work by recording instances of contact and contact attempts. In areas with a high rate of turnover among site staff, practitioners used tools to track staff as they move to new jobs in new sites or to identify new leads. Because PHIMS does not allow practitioners to easily move staff between sites, some LHDs created their own spreadsheets that enabled them to track this information themselves. Similarly, because PHIMS only tracks implementation information at the site level, informal tools capture *implementation strategies used across multiple sites*, for example, LHD-wide training sessions or the distribution of educational aides that help multiple sites simultaneously progress towards implementation target achievement*.*

#### Monitoring progress towards target achievement

We observed a variety of informal tools used in seven LHDs to monitor sites’ progress towards meeting program targets. Informal tools—particularly spreadsheets and templates—were used in conjunction with PHIMS. Practitioners download PHIMS data into spreadsheets to filter for specific information to *track implementation progress in each site*. This information enabled practitioners to create implementation strategies based on specific criteria, such as sites that are overachieving to serve as case studies or sites that are just under the achievement goal whose improvement could help practitioners meet overall KPI targets.

Practitioners used informal tools to capture *contextual information about sites’ progress towards meeting targets.* Using checklists and templates, practitioners collected detailed information about site-level barriers and facilitators to implementation, e.g., site priorities that might conflict or overlap with efforts to achieve program targets and reminders of contextual information that helps them support sites. Emails were a common tool employed by several LHD teams to document site visits and identify a plan of action, thereby creating a shared record between themselves and the contact for future use.

Informal tools aided collection of additional information about sites that are not required for state-level reporting but is helpful for *tracking LHD-level targets in addition to state-level targets.* One team maintained a spreadsheet that is separate to PHIMS but contains similar and overlapping information. This enabled the team to track their progress against a local equity framework which ensures that teams are attending to schools in high-need areas.

#### Guiding and troubleshooting PHIMS use

Bespoke informal tools were created by teams to enable them to use PHIMS better, for instance, by developing a consistent team approach to data entry. Teams developed tip sheets and guides to *facilitate the accuracy and consistency of data entered in PHIMS.* One participant developed an audit tool to guide and review the qualitative information entered into the system.

The timing of site visits and of data entry is an important component of HCI implementation. KPI data is reported quarterly, there are expectations regarding how often practitioners should meet with sites, and school calendars require practitioners to plan around school holidays when contacts are away. PHIMS has a scheduled reminder function but the users had limited control over when deadlines occur. So practitioners used informal tools to *plan for the deadlines that are set in PHIMS*, that is, deadlines to report KPI progress or complete a “scheduled follow-up visit” with a site. These tools ranged from simple reminders and task lists, to elaborate a spreadsheet that users adapted to their needs. Teams similarly printed hard copies of critical information from PHIMS and other electronic sources to keep at hand for *critical information needed “at a glance”.* Spreadsheets, checklists, and information sheets were observed hanging in many offices and cubicles containing information about timelines, priority lists, and reminders about when to follow up with key contacts.

Tools are used by teams to create backups of important data to *safeguard against PHIMS going offline or losing data*. This concern is based on known problems with PHIMS, namely periods of planned or unexpected server outages and difficulties in finding or “losing” data in PHIMS. Keeping backups reflected a mistrust of the formal, standardized system, including confusion about how it works, where data goes, and anxiety around losing data if changes are made. However, keeping data in multiple places also posed problems for ensuring data quality. One practitioner discussed potential “measurement error” due to mistakes in transposing data across multiple systems.

I ask [Name removed] if she has other systems to record data, and she tells me, “[name] is a spreadsheet queen, that’s my gripe with her. Everything’s recorded in about 7 different places [this is said as a platitude]. Which is irritating because to me I just see measurement error. Like, screaming at me because sometimes you forget to put it there or you miss it there and then it gets transferred or someone saves it incorrectly. I think it stems because PHIMS came in a few years ago and had a lot of teething problems and they kept missing or losing data.” —Field note from LHD E

#### Supporting teamwork

Using informal tools to *create detailed histories of work with sites* alleviated another concern: transferring knowledge to future practitioners in the case of potential turnover so that knowledge about a site does not reside with only one practitioner. Teams also use other tools to *strategize together, create plans, and coordinate activities*. Having a variety of records available to the whole team via electronic or physical filing systems enabled practitioners to create knowledge together by sharing resources and information about sites with one another and by collaborating to share and create strategies to address barriers.

#### Conducting evaluation

Conducting evaluation is a key competency in health promotion but not required of HCI staff at the local level. However, many practitioners performed evaluation activities to develop knowledge to help guide their approach to HCI. Informal tools, primarily online survey tools, were used to conduct *formative evaluations* to gain new insights about needs across sites. This information informed the design of educational aides, training, and other activities to support sites and increase KPIs. Practitioners sometimes entered *evaluation* data from workshops and events into online survey tools which enable them to easily analyze, tabulate, and report results.

#### Recording work that does not count towards HCI implementation

The final function we identified is that informal tools capture work that is outside the scope of HCI implementation. Teams used informal tools to *document their interaction with non-HCI sites and community organizations*. One team created a spreadsheet to collect information about new childcare businesses opening in the area. This team would work to support these sites but only enroll them in PHIMS after they agreed to participate in the formal program.

## Discussion

By examining PHIMS use in practice and the informal tools that arose alongside, we demonstrate the kind of information that practitioners value for implementation. While we observed that practitioners valued information to help them achieve HCI goals, we also observed that they valued information reflective of broader competencies and values of health promotion practice [28]—e.g., evaluation, teamwork, succession planning, innovation, respectful documentation of practice, and partnership building. Informal tools, therefore, served a range of important practice functions that enhanced both efficiency and flexibility of HCI work and provided functions not accommodated by PHIMS. These informal systems largely did not displace PHIMS; rather, they appeared to be complementary by enabling teamwork and integration of PHIMS into practice routines.

### Knowledge for implementation

We found practitioners and teams drew from a variety of tools and technologies outside of a standardized IT information management system to implement HCI. While in some instances, informal tools served as “workarounds” to limitations of PHIMS, their use went beyond that. By drawing on informal tools, practitioners generated information about trends and patterns of implementation across sites. Informal tools enabled practitioners to generate new knowledge about effective and efficient context-specific strategies to meet implementation targets. This finding reflects previous studies that illustrate that users who can access multiple types of technology use them congruently and concurrently to aide in practice and production of knowledge [29]. This is because most learning in organizational settings is informal, and thereby, facilitated by access to a variety of informal technologies [30, 31].

Informal tools provide a low-risk and low-investment option for experimenting with knowledge creation, whereas even simple design changes to standardized IT systems can be costly. In our study, practitioners used information recorded in informal tools to reflect on and make sense of their practice experiences, creating new knowledge about relationships and implementation that were eventually translated into data for the standardized PHIMS system. Templates and handwritten notes provided an efficient way to capture impressions that might change over the course of single or subsequent interactions or to track emergent barriers that may or may not prove influential to implementation. If the information captured via these systems turns out to be irrelevant, it can be easily changed or discarded. Conversely, information entered into standardized systems becomes permanently codified in the system and may be difficult to amend should impressions prove inaccurate or information proves meaningless [32]. The imperative of delineating types of knowledge and how to capture it is particularly relevant in contexts where personal and professional realms are blurred. Informal tools enabled practitioners to straddle the line between personal and professional. For instance, practitioners used handwritten notes to create memory aids for future interactions, while simultaneously maintaining a degree of privacy and discretion over the information they chose to input into PHIMS.

### Implementation and design of PHIMS

Some may argue that the use of informal tools alongside PHIMS could indicate that PHIMS is not fulfilling its intended purpose well enough. This position reflects an implementation science perspective in which the process of scale and spread is conceptualized as sequential and structured. In this perspective, IT systems enable standardization and replication of core components across sites. But our findings are better interpreted through a complexity lens [33]. Complexity theory posits that complex systems (made up of things, people, and process) are dynamic—constantly adapting in response to changes in context. That practitioners are using alternate systems with, and sometimes instead of, PHIMS is an example of users adapting and modifying technologies in sometimes unexpected, but retrospectively understandable, ways.

From its inception, the vision for PHIMS was a system that could support teams in coordinating implementation in addition to data collection for monitoring and reporting purposes. The designers adopted user-centered design principles and undertook a lengthy consultation process. However, this process was undertaken almost 5 years ago. It is important to note that PHIMS is “living” and has been adapted and updated. But this study provides deeper insights that may signal the extent to which the broader system of HCI implementation, including what the program is and what it takes to implement it, has changed over time. The functions we identify invite reflection on how the vision for PHIMS might also adapt to better support the increasingly sophisticated knowledge being developed by practitioners about HCI implementation. There are current existing mechanisms that could be strengthened to improve user feedback to inform adaptations to both PHIMS and its broader operating environment, i.e., in what ways it is used to support HCI. In addition, the results of this partnership research project are contributing insights for PHIMS system improvement (Conte, KP and Davidson, S. Using a ‘rich picture’ to facilitate systems thinking in research coproduction. In submission). Fully embracing the view that IT system development is an ongoing, iterative process that persists after initial design and implementation will be critical to ensuring its ongoing relevance and sustainability [34]. While recording systems have traditionally been used to track standardized key components of practice [9], computer technologies are becoming more responsive and better supporting user-defined adaptations. As electronic monitoring systems continue to develop, there is potential to design them to better capture emerging knowledge and innovations.

The use of informal systems also invites reflection on the appropriate delineation of the information that needs to be standardized and codified via an institutional IT system versus the type of information that should remain for local use only. It is likely infeasible to legitimately standardize implementation processes within a formal IT system across such a large geographical area with very different organizational arrangements and numbers of sites. Local nuances and variations in implementation approaches pose meaningful differences in practice that could be restrained through attempts at standardization. Further, formal large-scale IT systems like PHIMS have multiple users whose conflicting work processes often collide via the technology. For example, the value of seemingly tedious data entry may be unclear to users on the front lines of practice but may be justifiable to administrators responsible for the overall delivery and ensuring future funding [35, 36]. These issues are an invitation to resist over-standardization and to recognize the infeasibility of formal systems to fully anticipate and meet the needs of all possible users. Shifting contexts and information needs will require nimbleness, and informal systems allow for that. Formal IT systems may only need to serve a few key purposes well (e.g., monitoring and/or knowledge sharing) while allowing secondary functions to be added depending on need and purpose.

#### Limitations

It is likely that we were unable to capture the full range and instances of informal tools and systems that were used alongside PHIMS in practice. Ethnography is an opportunistic method which means that the data captured is dependent on what researchers have an opportunity to see or are allowed to see. The degree to which we obtained access varied across sites and precluded an in-depth analysis of contextual factors that might explain or predict the adoption of informal tools. We were less interested in documenting the prevalence of informal systems in practice, instead of exploring what it means that they are used at all and for what purposes. We are unable to fully appreciate whether the use of informal tools constitutes a threat to PHIMS use and sustainability. Future questions to ask are perhaps are informal tools a problem; whose problem are they; and if they are not a problem now, when might they become one? Our responsibility is to promote the dialog to address these issues. Another interpretation is that informal systems may support formal systems and thus help explain why PHIMS has been sustained while other similar systems have been abandoned [37, 38]. PHIMS is, in essence, a process or bundle of activities. The informal tools reflect practitioners’ dedication to practice, a value-add.

## Conclusion

Based on our observations, we conclude that PHIMS is serving important practice functions: the coordination, standardized reporting, and tracking of the large-scale implementation of a prevention program—something that, to our knowledge, has never been done at a large scale in population-level prevention. PHIMS may be doing less well at fulfilling its purpose as a practice-support system than intended, but it may be that doing *well enough* is the key to its success. We have also shown that while implementers use PHIMS to aide implementation, they also use informal tools to generate new knowledge that supports program delivery and quality practice. This knowledge complements standardized monitoring systems by enabling knowledge generation about implementation while supporting the integration of standardized systems to monitor KPIs. The challenge is to strike the right balance between the two: allowing for flexibility without over-standardizing practice.

## Supplementary information

**Additional file 1.** Additional information as per the consolidated criteria for reporting qualitative research (COREQ) checklist.

## Data Availability

The datasets generated and/or analyzed during the current study are not publicly available due information that could compromise research participant consent and privacy but can be made available from the guaranteeing author (PH) subject to appropriate precautions and upon reasonable request.

## Abbreviations

HCI: Healthy Children Initiative
IT: Information technology
KPIs: Key performance indicators
LHDs: Local health districts
NSW: New South Wales
PHIMS: The Population Health Information Management System

## Footnotes

1 Note: There were 15 LHDs in New South Wales at the time of the study. One team delivered HCI programs in two LHDs.
