# Supplementary material for: Capturing implementation knowledge: applying focused ethnography to study how implementers generate and manage knowledge in the scale-up of obesity prevention programs
Source: Implement Sci. 2019 Sep 18;14:91. doi: 10.1186/s13012-019-0938-7 (PMC6751600; doi:10.1186/s13012-019-0938-7)
Supplement: Supplementary file 1 — Additional file 1. Additional information as per the consolidated criteria for reporting qualitative research (COREQ) checklist. [file 13012_2019_938_MOESM1_ESM.docx]

| **Additional File 1. Additional information as per the consolidated criteria for reporting qualitative research (COREQ) checklist** | |
| --- | --- |
| **Domain 1: Research Team and reflexivity** | |
| **Personal Characteristics**   1. Interviewer/facilitator; 2. Credentials; 3. Occupation; 4. Gender; Experience and Training | Ethnographic data collection was conducted by KC, VL and SGR. KC is a public health researcher with 7 years experience and training in qualitative methods, and 11 years experience as a health promotion practitioner. She trained the other staff members and oversaw the fieldwork activities. VL and SGR are trained ethnographers who collectively have 26 years experience. All were employed as research fellows by the University of Sydney, in roles funded by the Australian Prevention Partnership Centre - a collaboratively funded research centre that receives funds from the NHMRC and others. AS was an undergraduate student intern who had previous experience working with electronic health recording systems. She completed a 6-month internship under KC’s supervision during which she conducted the initial analysis. Relevant to this research, the NSW Department of Health –whose employees are co-authors of this paper- is a funding partner of TAPPC. PH oversaw all study activities as guarantor. She is a former health promotion practitioner in NSW and public health researcher of 30+ years |
| **Relationship with participants**  6. Relationships established; 7. Participant knowledge of the interviewer; 8. Interviewer characteristics | The process of synthesising the research design and study questions involved getting to know many of the research participants and consulting them as part of the design process. This design work took place primarily in the early phase of fieldwork conducted by KC, one year prior to the in-depth ethnographic work on which this analysis is based. Ethnographic fieldwork also included many informal interactions like lunch, coffee, car trips that enabled more intimate relationships and personal discussions with researchers, beyond usual interview settings. Participants were informed that the researchers were working in partnership with decision-makers at the NSW Ministry of Health. To ensure confidentiality, they were reassured that only the researchers would have access to and view de-identified quotes, and that interpretations of data would be shared with them for feedback prior to presentation to the Ministry. |
| **Doman 2: study design** | |
| **Theoretical framework**  9. Methodological orientation and theory; | This study was co-designed with partners at the NSW Ministry of Health who informed on the research approach and questions. The theoretical grounding of the overall study is outlined in detail in another paper^1^. The methodological orientation was primarily ethnographic. |
| **Participant selection**  10. Sampling; 11. Method of approach; 12; Sample Size; 13. Non-Participation | At the site-level, all teams that implemented the Healthy Children’s Initiative agreed to participate. At the team level, one-hundred and six individuals agreed to participate of one-hundred and twenty-six identified individuals. Our sampling was opportunistic in that we only observed individuals that were present at the time of the observation, so some of the non-participants may not have been present and observed. No HCI team member declined participation, but several did not return their consent forms. KC met with most sites in-person or via phone to discuss the study purpose prior to fieldwork, and followed up with written documentation of study information and consent. At the time of fieldwork, researchers discussed the study objectives with participants and answered questions. Individuals had the opportunity to provide us with their decision to consent privately. |
| **Setting**  14. Setting of data collection; 15. Presence of non-participants; 16. Description of sample | Data collection occurred in multiple places including at the local health district’s offices, riding in cars to site visits, at site visits, at meetings and via phone. Fieldwork visits ranged from 1-5 days. We informed non-participants about the research and that we were documenting our observations, and assured them we would not collect any detailed information about them. |
| **Data collection**  17. Interview guide; 18. Repeat interviews; 19. Audio/visual recording; 20. Field notes; 21. Duration; 22. Data saturation; 23. Transcripts returned | KC developed a field note template and revised it in collaboration with SG and VL to create a structured debriefing process to ensure consistency among researchers. Extensive field notes were recorded as soon as possible following the fieldwork. On occasion, and when granted permission, we audio recorded brief interviews or team meetings. These recordings were transcribed verbatim by a third party and checked for accuracy. Given the nature of our approach, obtaining data saturation was not possible. Rather, by including all sites we ensured the widest range of possible experiences and viewpoint. Except for a few instances to double-check our interpretations, we did not share our field notes with participants. |
| **Domain 3. Analysis and findings** | |
| **Data analysis**  24. Number of data coders; 25. Description of the coding tree; Derivation of themes; 27. Software; 28. Participant checking | KC, VL and SG developed an initial coding structure and conducted coding for all field notes; each was coded by two researchers in NVIVO qualitative data management software^2^. From this initial codebook, a subsequent and more detailed coding analysis for this paper was conducted by AS, guided by KC. KC and AS discussed emerging themes on an ongoing basis with VL, SG, and PH, and ideas were consolidated and presented to the wider team for feedback. The coding and theming process is discussed in detail in our manuscript. We disseminated a findings brief to participants, and hosted 2 webinars to present and discuss interpretations. |
| **Reporting**  29. Quotations presented; 30. Data and findings consistent; 31. Clarity of major themes; 32. Clarity of minor themes | We report our results using quotes extracted from the field notes that are illustrative of both major and minor themes. Consistency between data and findings were iteratively checked throughout the analysis, to ensure that consistency and accuracy between the two. |

1. Conte KP, Groen S, Loblay V, et al. Dynamics behind the scale up of evidence-based obesity prevention: protocol for a multi-site case study of an electronic implementation monitoring system in health promotion practice. Imp Sci. 2017;12(1):146.

2. Nvivo qualitative data analysis software. 11 ed: QSR International Pty Ltd.; 2015.
